# Second order and transverse flow visualization through three-dimensional particle image velocimetry in millimetric ducts

**DOI:** 10.1016/j.expthermflusci.2024.111296

**Published:** 2024-12

**Authors:** N.C. Harte, D. Obrist, M. Versluis, E. Groot Jebbink, M. Caversaccio, W. Wimmer, G. Lajoinie

**Affiliations:** a Department of Otorhinolaryngology, TUM School of Medicine, Klinikum rechts der Isar, Technical University of Munich, Munich, Germany; b ARTORG Center for Biomedical Engineering Research, University of Bern, Bern, Switzerland; c Physics of Fluids Group, Max Planck Center for Complex Fluid Dynamics, Faculty of Science and Technology, Technical Medical (TechMed) Center, University of Twente, Enschede, The Netherlands; d Multi-Modality Medical Imaging Group, Faculty of Science and Technology, Technical Medical (TechMed) Center, University of Twente, Enschede, The Netherlands; e Department of Otorhinolaryngology, Head and Neck Surgery, Inselspital, Bern University Hospital, Bern, Switzerland

**Keywords:** Scanning particle image velocimetry (PIV), Three-dimensional three-component (3D3C), Secondary flow, Microfluidics, Computational fluid dynamics (CFD), Dean vortices, Low Reynolds number

## Abstract

Despite recent advances in 3D particle image velocimetry (PIV), challenges remain in measuring small-scale 3D flows, in particular flows with large dynamic range. This study presents a scanning 3D-PIV system tailored for oscillatory flows, capable of resolving transverse flows less than a percent of the axial flow amplitude. The system was applied to visualize transverse flows in millimetric straight, toroidal, and twisted ducts. Two PIV analysis techniques, stroboscopic and semi-Lagrangian PIV, enable the quantification of net motion as well as time-resolved axial and transverse velocities. The experimental results closely align with computational fluid dynamics (CFD) simulations performed in a digitized representation of the experimental model. The proposed method allows the examination of periodic flows in systems down to microscopic scale and is particularly well-suited for applications that cannot be scaled up due to their complex, multi-physics nature.

## Highlights

• 3D scanning Particle Image Velocimetry system for measuring oscillating flows.
• Stroboscopic and semi-Lagrangian Particle Image Velocimetry analysis.
• Net motion and time-resolved flow quantification with two analysis methods.
• Validation of experimental data with computational fluid dynamics simulations.
• Effect of geometry on transverse flow in millimetric ducts.

## 1 Introduction

Flow related problems are ubiquitous in technical, industrial, and biological applications. In industry, the efficiency of chemical reactors [1], the success of pharmaceutical (e.g., vaccines) production [2], [3], as well as food and beverage processing [4], [5] heavily rely on well-controlled flows. Maritime transport is eminently linked to fluid dynamics [6], [7]. In medicine, flow is central to the development of cardiovascular disease [8], [9], [10], [11] and the study of lung diseases in fluidic microchips [12]. Flow is also crucial in understanding the function of organs such as the cochlea [13], [14], which is not yet fully understood [15].

Simulations are not always possible for complex geometries because it can be difficult to model an accurate representation of the shape and generate a high-quality mesh. In addition, setting the correct flow boundary conditions can be extremely challenging, in particular in a biological context. The flows therein involve fluid–structure interactions, where the viscoelastic properties of the structure are largely unknown. Furthermore, these flow problems are often multiscale, which results in complex numerical models with high computational costs which, ultimately, must also be validated experimentally.

The most widely used flow quantification techniques are probably particle image velocimetry (PIV) and particle tracking velocimetry (PTV), which both involve tracer particles. PIV is based on the cross-correlation of sub-matrices in successive images, while PTV consists in tracking the trajectory of individual particles [16]. Other flow visualization techniques include Schlieren photography and shadowgraphy, both exploiting the variations in the refractive index of the fluid of interest, e.g., due to temperature or density variations [17], [18]. Finally, techniques were developed that use laser-induced fluorescence with fluorescein dye to visualize, e.g., secondary flow vortices [19], [20]. In their vast majority, these techniques are designed to image 2D slices of the flow. Although this may be sufficient in some cases, most flow phenomena are intrinsically three-dimensional and thus require 3D visualization.

This led to the development of several PTV and PIV techniques to measure the full velocity field in 3D. They are referred to as three-dimensional three-component (3D3C) measurements. 3D-PTV requires at least two imaging directions (in practice up to 5) to determine the out-of-plane velocity. The technique is effective, but experimentally complex and costly, and the required processing is computationally expensive. 3D-PTV has been adapted to be feasible with a single camera [22], which, however, requires a more complex calibration, and increases the sparsity requirement. 3D-PIV is not limited by sparsity, which improves the spatial coverage – required for complex flows – and/or reduces the necessary acquisition time. Several variations of this technique have been proposed, such as holographic PIV, tomographic PIV, and scanning PIV. The spatial resolution of digital holographic PIV is limited mostly by the image sensor (i.e., CCD) [16], [23]. In tomographic PIV, the whole test volume is illuminated at the same time [24]. Tomographic PIV provides high spatial and temporal resolution, but requires substantial pulse energies [16]. In scanning PIV, fast successive scans of a laser sheet through a volume of interest are recorded with a large depth of field by a single synchronized high-speed camera [25], [26]. Hori and Sakakibara [27] applied a scanning laser sheet with stereoscopic PIV and a dual camera setup to measure the 3D3C velocity field. Scanning comes at the cost of higher camera frame rates to achieve the desired temporal resolution. The advantage is a high accuracy, which is mainly limited by the camera technology. As cameras continue to improve in resolution, sensitivity, and frame rate, the importance of scanning PIV is also growing.

**Fig. 1:**
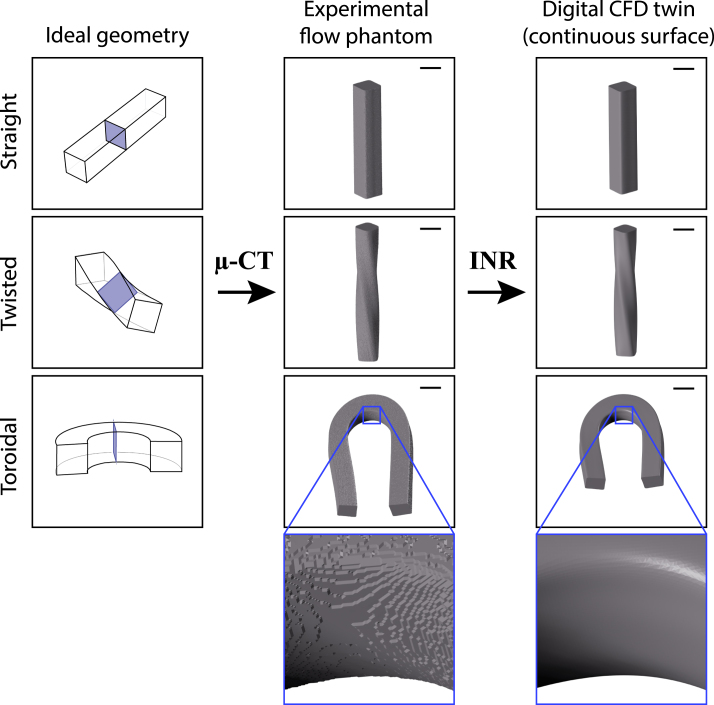
Flow channels of the straight, toroidal and twisted samples. From left to right: idealized CFD models with a $2\times 2\;{\text{mm}}^{2}$ cross-section, µ-CT scans of the measurement samples, and CFD models obtained with continuous implicit neural representations (INR) of the scanned samples [21]. The scale bar represents 2mm.

Two aspects remain challenging with 3D-PIV, which are small dimensions, i.e., 3D-microPIV, and multiscale flows. The former is a consequence of the optics used for small-scale imaging, as they typically provide a very limited depth of field. Multiscale problems arise when studying weak flow features within a stronger flow, especially when these features are orthogonal to the main flow, as is the case for Dean cells, for example. Secondary or transverse flows are of particular interest in the inner ear, where they contribute to steady streaming [28], [29] and are suspected to play a role in hearing physiology [14], [30]. Transverse flow is also gaining attention for helical stents in the superficial femoral artery as a possible mechanism to improve stent patency [31], [32]. Several studies have measured transverse flows in toroidal geometries using PIV to investigate their importance for the arterial vessel system [33], [34], [35], [36], [37]. The difficulty of measuring transverse flows lies in the fact that they are several orders of magnitudes smaller than the axial flow, especially at low Reynolds numbers. In addition to scanning PIV, digital holographic PIV and tomographic PIV can potentially be used to measure minute transverse flows. These techniques are, in particular, applied to study turbulent flows [38], [39] and droplets [40], [41]. Scanning PIV offers similar capabilities for the frequencies of interest and is more user-friendly as it requires a less complex experimental setup.

Here, we present the development and experimental validation of a scanning 3D-PIV system for small-scale oscillatory flows, which is capable of resolving transverse flows that are up to two orders of magnitude weaker than the main axial flow. With this setup, we experimentally investigate the effect of torsion and curvature on transverse flows in three millimetric geometries: a straight square duct, a toroidal duct, and a twisted square duct. The measured flow oscillation frequencies range from 5 to 20Hz. The setup is accompanied by two original and complementary forms of PIV analyses: stroboscopic and semi-Lagrangian, which allow to quantify the net flows and time-resolved axial and transverse velocities in the same dataset. The toroidal duct, only exhibiting curvature, features pairs of counter-rotating flow cells oriented in the transverse plane, known as Dean cells [42], [43]. Inside twisted straight ducts, which only contain torsion, saddle flow patterns arise, which strongly depend on the cross-section of the duct [44], [45], [46]. The respective time-averaged net motions show rotating cells, which are of interest for cross-sectional mixing and mass transport in biomedical and industrial microfluidic applications. The duct shapes were also measured using micro-CT (µ-CT), and used to perform geometry-specific CFD simulations to validate the experimental results. We have further compared these results to CFD simulations with idealized square cross-sections [47], which evidences the key importance of the exact geometry for the flow in these transverse planes.

**Fig. 2:**
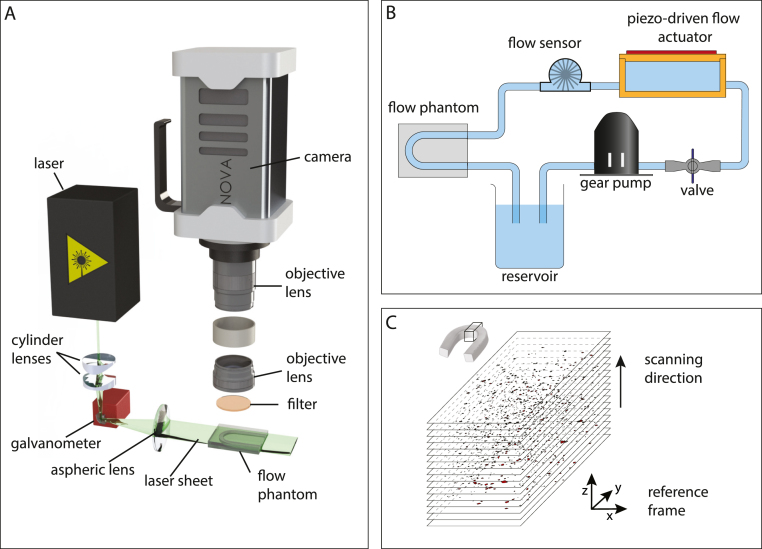
(A) Schematics of the optical configuration at the core of the 3D-PIV measurement setup. The laser sheet is shaped by two cylindrical lenses that focus the laser on the galvanometer mirror. A lens with its focal point on the mirror generates a straight laser sheet, which remains, at all times, parallel to the xy-plane. In the z-direction, the sheet is focused onto the sample to minimize the laser sheet thickness. (B) Flow system used to drive the flow through the flow phantom. The oscillatory flow is driven by a piezo-actuated pump. A gear pump creates a steady flow between measurements to prevent the sedimentation of the fluorescent tracer particles. A valve prevents any steady flow during the measurements. (C) 3D particle volume formed by stacking the particle images of a single measurement scan (colors inverted). An animation of the raw data can be found in the supplementary material.

## 2 Material and methods

### 2.1 Measurement setup

#### 2.1.1 Models

We measured transverse flows within straight, toroidal, and twisted flow channels (Fig. 1), which represent the main deformations of the square duct, and the shapes in which transverse flows have been simulated [47], [48]. The toroidal flow channel has a radius of curvature of approximately 2.8mm, which corresponds to a curvature of $\kappa =0.36\;{\text{mm}}^{-1}$. The torsion of the twisted flow channel is approximately $\tau =1/6\;{\text{mm}}^{-1}$ in the flow phantom. These values are within the range proposed by Viergever [49] and match values observed in human cochleae [50], [51]. The flow channels were created by heating 2mm acrylonitrile–butadiene–styrene (ABS) square rods with a heat gun and mechanically bending them into the desired shape. The bent ABS rods were then cast in polydimethylsiloxane (PDMS). After curing, the ABS rods were dissolved with acetone and only the PDMS casting remained. A heat bath at 55 degrees Celsius was used to accelerate the dissolution process. The flow channels were extended at each end with a straight segment of at least 20mm length to minimize possible entry length effects from the inlet and outlet. The test sections have a length on the order of a centimeter, as shown in the micro-CT scans of Fig. 1. The recorded section, however, depends on the field of view of the camera which was approximately 2mm in our case. The details of the optical system will be discussed in Section 2.1.4. To perform computational fluid dynamics (CFD) simulations in the same geometric shapes as were used in the measurements, the samples were measured using a high-resolution µ-CT scanner (µCT, SCANCO Medical AG, Switzerland, isotropic voxel size of 20 µm). Using implicit neural representations [21], the flow channels were segmented from the µ-CT scans and converted into a continuous representation from which a CFD surface at any arbitrary precision can be derived. The scan and its representations are shown in Fig. 1 for the three models.

#### 2.1.2 Fluid solution

The density and viscosity of the perilymph in the cochlea are similar to those of water at body temperature [52]. We used an aqueous solution with 49 wt% urea to obtain a refractive index of about 1.41 (measured by [53] at 589nm), which matches the PDMS specimen. Refractive index matching is needed to avoid optical distortion. The density of the solution ρ=1133  kg/m^3^ and dynamic viscosity μ=1.7  m Pa s are higher than those of water [53], [54]. This composition, however, remains the best compromise known to the authors for optical refractive index matching to PDMS. Homogeneous fluorescent tracer particles (Fluoro-Max dry red fluorescent polymer microspheres from Thermo Scientific™) with a diameter of 6 µm and a density of 1050  kg/m^3^ were added to the solution.

#### 2.1.3 Flow system

Fig. 2A shows a schematic of the experimental setup. A custom-built actuator pump based on a piezoelectric element (PHUA6630; PiezoListen™ series) induced an oscillatory motion in the fluid during measurements. The actuator was driven by a Raspberry Pi 4B mini-computer and a Hifiberry AMP2 amplifier. The piezoelectric transducer was attached to a thin 3D-printed polyethylene terephthalate (PET) shell, which was mounted on a small, sealed fluid container. This piezo-driven flow actuator was used to induce the oscillatory flow through the flow phantoms during the measurements.

Between measurements, a continuous flow through the phantom was generated using a gear pump (12V, Kavan, type 0190.121; Kavan GmbH, Nurnberg, Germany). The only role of the gear pump was to prevent sedimentation of the tracer particles between measurements and to keep the particle suspension homogeneous. To be sure that the gear has no influence on the measurements, the flow through the pump was fully stopped by a manual valve positioned between gear pump and the piezo-driven pump so that only the latter was allowed to drive the oscillatory liquid flow through the flow phantoms during measurements. The flow rate and fluid temperature were monitored with a commercial thermal flow sensor (Sensirion® SLF3S-0600F) placed between the oscillatory pump and the specimen. The inner tube diameter in the flow circuit was 2mm. To minimize the system’s inertia and achieve higher flow rates, the fluid volume in the flow system was minimized to approximately 80 ml. Nonetheless, owing to the limited bandwidth of the flow system, velocities were lower for 20Hz than for 5 and 10Hz (Table 1).

#### 2.1.4 Optical system

The optical setup, shown in Fig. 2, uses a continuous wave frequency-doubled Nd:YAG(−532nm) laser to excite the fluorescent particles, which have an absorption maximum at 542nm. The laser beam was expanded into a sheet by three lenses. First, a cylindrical plano-concave lens diverges the laser beam in the x-direction, while a cylindrical plano-convex lens focuses it onto the rotating Galvanometer mirror (Saturn 1B Single Axis Galvo(56S), ScannerMAX). This Galvanometer is placed in the focal distance of a plano-convex aspheric lens, which collimates the beam in the desired sheet and focuses it in the elevation direction to minimize the sheet thickness within the camera’s depth of field. The long focal distance of the lens (f=121mm) minimizes the variations in the thickness of the sheet within the area of interest. This configuration also ensures that the laser sheet translates through the sample without rotation (Fig. 2B). To characterize the laser sheet, a glass plate coated with a thin layer of PDMS containing fluorescent Nile Red was fixed at a 45° angle to the laser and camera. The beam width was defined as the 1/e width of the profile after correction for the PDMS layer thickness. The laser sheet thickness was estimated as $\delta_{l}=13.5\pm 0.9$  µm. The details of the laser sheet calibration and the variation in sheet thickness through the sample are provided in Appendix A.1. The rotating mirror was driven by a sawtooth waveform, which provides a linear position of the laser sheet as a function of time (Fig. 2B). The scanning frequency and amplitude can be tuned independently through the driving frequency and voltage (see Appendix A.2 for more details).

A high-speed CMOS camera (NOVA S16, Photron, Tokyo, Japan), captured the fluorescence emitted by the tracer particles synchronized with the laser scanning. The measured test section had a volume about 1×  2×  2$\;{\text{mm}}^{3}$. We combined two commercial camera lenses front-to-front to obtain an optimum combination of magnification and depth of field. A tele-macro lens with a focal length f=105mm, was attached to the camera in the normal direction and focused to infinity. The aperture was closed as much as possible to achieve a large depth of field. A macro lens with f=12.5–75mm, was attached to the first lens in reversed direction and was also focused to infinity. The aperture and focal length of the second lens were adjusted to obtain the desired field of view. The diaphragms of the lenses enabled an arbitrary large depth of field which is a key feature of the system that allows high-aspect ratio measurements that are otherwise very challenging at this scale. Here, we ensure that the depth of field is sufficient through optical assessment at the proximal and distal imaging planes. We have used a frame rate of 20 kfps for 5Hz flow excitation and 40 kfps for 10Hz and 20Hz. A dichroic filter was placed between the lenses to remove the small fraction of laser light scattered by the sample. We calibrated the PIV system using a resolution target (1951 USAF). The spatial imaging resolution was approximately 5×5×15  µm for a typical focal length of f=22mm (see Appendix A.2).

#### 2.1.5 Experimental procedure

The specimen was aligned with the laser such that the sheet was focused on its center, which was achieved by first aligning the sample manually and then adjusting the offset voltage of the sawtooth signal driving the mechanical mirror. The measurement was only started when the flow sensor reported steady oscillations. A pulse delay generator triggered the waveform generator and the camera for data acquisition. The high-speed camera memory allowed the recording of 45 cycles, with 20 scans per cycle and a resolution of 256 × 496 pixels. For each scan, we recorded a volume consisting of 200 layers for actuation frequencies of 5Hz and 10Hz. To compensate for the increased frame rate necessary at higher actuation frequencies, we reduced the number of layers to 100 at 20Hz (Table 1). This increased the number of recorded cycles to 90. A resolution of 128 × 496 pixels doubles the number of recorded frames.

**Table 1:** Measurement parameters. The actuation frequency f refers to the fluid oscillation frequency, while $W_{0}$ denotes the velocity amplitude. The number of layers is given by the ratio of the camera frame rate to laser scan rate.

f (Hz)	Scan rate (Hz)	Frame rate (fps)	# layers	$W_{0}$ (mm/s)
5	100	20,000	200	4−8
10	200	40,000	200	4−7
20	400	40,000	100	3−6

We performed measurements in three samples (Fig. 1) for three actuation frequencies (5, 10, and 20Hz). The flow velocity amplitude was adjusted by changing the gain of the signal driving the piezoelectric actuator. For each frequency, we measured different flow velocity amplitudes in the operating ranges of the actuator pump. The Reynolds numbers (between 1 and 10) were close to the Stokes regime and therefore the fluid phenomena were laminar, almost creeping. We define the Womersley number as [47]: $\alpha =\frac{d}{2}\sqrt{2\pi f\rho /\mu }$ where d=2mm is the typical cross-sectional width of the channels, f is the driving frequency of the piezo-driven pump, ρ is the density of the fluid, and μ is the dynamic viscosity of the fluid. The resulting Womersley numbers (4.5 to 9) were above the quasi-steady regime (α>1).

### 2.2 Particle image velocimetry analysis

**Fig. 3:**
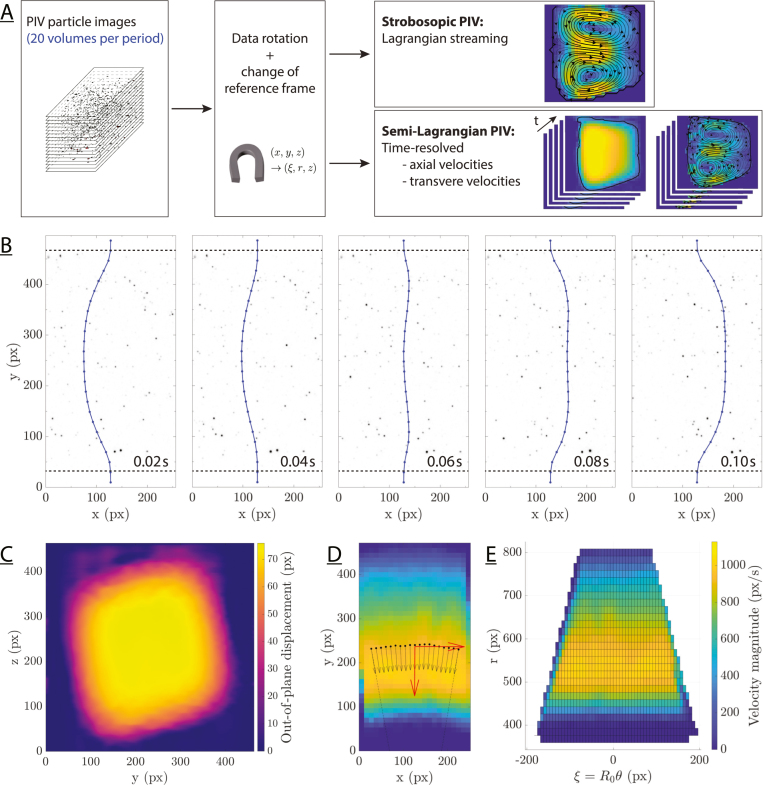
(A) The particle images were analyzed using the stroboscopic and the semi-Lagrangian PIV method. In both procedures, the data was rotated to align the axial flow direction with the x-axis. In the toroidal duct, the reference frame was changed from Cartesian (x,y,z) to polar coordinates (ξ,r,z) to define transverse planes of constant angles and to average velocities along the curved channel. The stroboscopic approach was used to quantify net motions, i.e., Lagrangian streaming, while the semi-Lagrangian PIV gave time-resolved velocities. (B) Reconstructed average particle displacement (blue line) in the central measurement plane for an oscillation frequency of 5Hz (colors inverted). The dotted line indicates the approximate duct boundaries. (C) Reconstructed average particle displacement amplitude in the transverse plane. (D) Velocity magnitude in Cartesian coordinates. Radial vectors are shown in black at the velocity-weighted y-positions (black dots). The red arrows indicate the radial and axial reference velocities. (E) Velocity magnitude in polar coordinates, interpolated to a rectangular grid.

We have developed two complementary analysis methods to quantify transverse velocities, which exploit the same measurement data: stroboscopic and semi-Lagrangian PIV (Fig. 3A). The stroboscopic approach shows the transverse net motion with high accuracy, while the semi-Lagrangian approach is used to visualize instantaneous, time-resolved transverse flows. In principle, an (Eulerian) net motion could be obtained from the time-resolved approach by averaging instantaneous velocities over time (Eulerian streaming). This approach, however, provides a much lower signal-to-noise ratio (SNR) than the stroboscopic approach for net motion. The stroboscopic approach provides a Lagrangian mean velocity, representing the mean displacement of a particle during one oscillation cycle. An animation showing volumetric measurement data (color inverted) is provided in the supplemental material as an example. The analysis is based on two assumptions. First, we consider the scanning rates to be high enough to neglect the motion occurring between the frames forming a single volume. Second, to increase the SNR, we assume that the velocities can be averaged along the channel axis since we measure a short test section (0.5–1mm along the axial direction).

#### 2.2.1 Alignment, data rotation, and PIV settings

Moving linearly from top to bottom, the scanning laser sheet illuminates a narrow section of the flow channel. The scanning direction defines the z-axis. Consequently, the planes captured by the camera are parallel to the xy-plane, i.e., the measurement plane. The x-axis is aligned with the channel axis. Since we aim at measuring minute flows orthogonal to the channel axis, it is necessary to ensure that this channel axis is perfectly aligned with the x-axis as defined for the analysis. Such precision cannot be achieved experimentally, and the orientation of the data has to be adjusted in a processing step. To that end, the angle between the x-axis of the camera and that of the main axial flow is determined from a first PIV analysis performed in the xy-plane (which is also the plane of the laser sheet and that of the raw images recorded by the camera) at mid-height of the measurement volume. The angle is calculated from the median of the angles of the velocity vectors. The volumes (4D stack of images) are then rotated by this angle to minimize the lateral velocity component v corresponding to the y-direction. This step facilitates both the stroboscopic analysis where it avoids blurring the flow patterns when averaging in the x-direction, and the semi-Lagrangian analysis where now only the x-component of the velocity u has to be considered. For simplicity, we write the velocities in the x-, y-, and z-directions as u, v and w.

The image intensity of each layer is normalized with respect to its maximal intensity excluding the top percentile for robustness (averaged over 15 images in time) to compensate for the brightness reduction in layers that are physically further away from the camera. The PIV analysis is based on PIVlab [55], [56], a particle image velocimetry tool for MATLAB®, where we use the built-in multi-pass technique with decreasing interrogation window sizes: 64 × 64, 32 × 32, 22 × 22 pixels and a 50% overlap of the interrogation areas. While we apply these three passes in the measurement plane (xy-plane), only two passes (with final pass size of 32 × 32 pixels) are used in the orthogonal planes (e.g., in the xz- and yz-plane), which are constructed through interpolation from the measured slices, and have a lower resolution. The spatial resolution of the resulting 3D velocity field is approximately 80  µm. The velocities are collected in 20 bins per cycle, and the median velocity over all cycles that could be recorded by the camera is used to increase the SNR.

#### 2.2.2 Stroboscopic PIV

The stroboscopic PIV method provides a fast way to analyze the particle images in order to obtain the quantitative net flow field (Lagrangian mean velocity). Here, we use the 3D volumes constructed as explained in Section 2.2.1. In short, the volumes are reconstructed by stacking the particle images corresponding to a single scanning period and rotating the resulting volume so as to align it with the axial flow direction. In this stroboscopic PIV procedure, we avoid computationally expensive 3D PIV analysis by performing the PIV analysis in 2D, directly in the yz-planes which are orthogonal to the channel axis. We call this analysis stroboscopic because the aforementioned PIV processing is performed on image pairs separated by exactly 1 oscillation cycle, thereby cancelling the periodic motion and leaving only the net motion.

To prepare the yz particle images, the 3D volume is first cubically interpolated in the z-direction to obtain an isotropic resolution, i.e., the same resolution in the x-, y- and z-directions. We then construct artificial particle images corresponding to the yz-plane by averaging the particle volume in the x-direction over 21 pixels. This step guarantees sufficient particle density for 2D PIV analysis, and sufficient particle persistence. Indeed, any axial net movement of more than the pixel size (i.e., 5μm) would otherwise cause the particle to disappear between the two images compared by PIV. Note that this averaging step does not compromise precision since the flow features in the axial flow direction are much larger than the 21 pixels (∼100μm) used for averaging. Adjacent frames are constructed using the exact same procedure with 50% overlap between the averaged volumes. The initial volume for the series is chosen at the peak axial velocity, corresponding to a zero displacement of the particles. Note that this choice is arbitrary. The PIV analysis performed in these artificial, transverse particle images then provides the net transverse velocities $v_{\text{net}}$ and $w_{\text{net}}$ in the y- and z-directions, respectively. To increase the SNR, we average $v_{\text{net}}$ and $w_{\text{net}}$ over all periods and over all transverse planes. The resulting velocity field is the average net velocity in the transverse plane.

#### 2.2.3 Semi-Lagrangian PIV

The semi-Lagrangian PIV approach is a 2-step process. First, we measure and compensate for the particle displacement in the axial direction to construct the “semi-Lagrangian” transverse planes. In a second step, particle images are constructed from these Lagrangian planes and analyzed pairwise using PIV. The particle volumes are prepared as explained in Section 2.2.1. In the first step, we perform a PIV analysis on the particle image recorded by the cameras, i.e., in the xy-plane that are co-planar with the laser sheet. This provides the velocity components u and v in the x- and y-directions, respectively. The lateral velocity component v is already minimized directly during the preparation of the 3D volumes. Then, a first PIV analysis is used to compute the angle between the x-direction of the camera and the axial flow direction. The volumes are then rotated by this angle (see Section 2.2.1). Thus, only u must be compensated for in this semi-Lagrangian analysis. To that end, u is averaged along the x-direction and subsequently integrated over time to obtain the axial (main flow) displacement. Fig. 3B shows an example of the displacement recovered in the central layer of the volume, i.e., at mid-height. Repeating the procedure for each xy-plane provides the main axial displacement in the full yz-plane as a function of time, as can be seen in Fig. 3C, for one time-step.

For the second step, the measured axial displacements are first interpolated using a spline function to match the initial resolution of the particle volumes in the y- and z-directions. Because these semi-Lagrangian displacements allow for tracking particles in the main axial flow (see, for example, the blue line in Fig. 3B), they can be used for constructing particle images in which the axial oscillatory motion is compensated for. The first frame of the image sequence for PIV analysis consists of a yz-cross-section of the 3D particle volume. Each successive particle image is constructed by extracting the pixels closest to the computed Lagrangian displacement within the 3D particle volume (at the time-step considered) and assembling them into an artificial frame. For the same reasons of particle density and persistence as explained in Section 2.2.2, the pixels of these new particle images consist of an average over 11 volume voxels in the x-direction. Adjacent particle images are constructed by repeating the procedure for different initial yz-cross-sections of the 3D volume so that the resulting images have a 50% overlap (i.e., 5 pixels of the raw particle image). Pairwise PIV analysis on these new particle images then provides the transverse velocities v and w in the y- and z-directions, respectively. Finally, we average the transverse velocities over all cycles and yz-planes. For the toroidal duct, the same approach is used on the radial velocity $u_{r}$ instead of v after projection in polar coordinates, see Section 2.2.4.

#### 2.2.4 Frame of reference for the toroidal duct

In the toroidal duct, the axial and radial velocity components are not perfectly aligned with the x-axis and y-axis because of the curvature of the channel. To account for the deviation, we computed the geometrical center of curvature and used it to define a local polar coordinate system. The u and v velocities are decomposed in the local coordinate system to obtain an angular velocity $u_{\xi }$ and a radial velocity $u_{r}$, while the third velocity component is left unchanged and $u_{z}=w$.

More specifically, we first compute the center of curvature by performing a PIV analysis to determine the in-plane velocity field for each layer. The velocities are averaged in the z-direction over these layers to better estimate the flow field. We then extract the locations of the strongest flow for each x-coordinate, by taking a velocity-weighted average of the y-coordinates, in the y-direction (Fig. 3D). We estimate mean velocities at these locations by averaging over 11 pixels in the y-direction. The normalized, in-plane reference velocity vector is defined as the median velocity over these locations (red arrows in Fig. 3D). To obtain more uniformly distributed mean vectors, we fitted the angles between the individual mean vectors and the reference vector. Finally, the (in-plane) center of curvature $R_{c}$ is determined as the point with the smallest mean distance to all normals on the mean velocity vectors. To optimize $R_{c}$, the angle between the reference velocity and the y-axis is varied. The optimal angle is chosen as the angle at which the transverse velocities summed over the yz-plane are minimal. To decompose the in-plane velocities, we defined at every grid point a radial unit vector ${\overset{ˆ}{e}}_{r}$ pointing towards the center of curvature $R_{c}$ and an angular unit vector ${\overset{ˆ}{e}}_{\xi }$. Subsequently, the velocity vectors are projected onto these angular and radial unit vectors. The radial velocity here corresponds to the transverse velocity in the xy-plane, while the angular velocity represents the axial velocity. Finally, the velocities are interpolated to a uniform grid of r and $\xi =R_{0}\theta$ (Fig. 3E). This transformation is then applied to each layer such that $\left(u,v,w\right)\;\to \;\left(u_{\xi },u_{r},u_{z}\right)$ and (x,y,z)→(ξ,r,z). The out-of-plane velocity w is obtained as explained in the semi-Lagrangian PIV in Section 2.2.3: $u_{z}=w$.

### 2.3 Computational fluid dynamics simulations

#### 2.3.1 Simulation models

We simulated fluid flow in the experimental geometries and in idealized geometries (Fig. 1). The idealized geometries were constructed using established methods [46], [57] with centerline curvature $\kappa =1/3\;{\text{mm}}^{-1}$ and torsion $\tau =1/8\;{\text{mm}}^{-1}$. To save computational resources, we chose a total arc length of the centerline of 10mm for all models. The model cross-section is 2mm×2mm. Note that the experimental models have slightly different curvature and torsion values and are not exactly square in cross-section because they were handcrafted. For the geometry-specific simulations, a CFD surface was generated from the continuous representation of the measurement models (Fig. 1).

#### 2.3.2 Numerical simulations

The flow was modeled as incompressible flow of a Newtonian fluid [52]. We used a Dirichlet pressure boundary condition which varied sinusoidally in time at the inlet and zero pressure at the outlet surface. No-slip boundary conditions were imposed on the walls. The fluid properties from Section 2.1.2 were used. The simulation parameters were set to match the measurements: we used oscillation frequencies of 5, 10, and 20Hz and extracted the mean axial velocity amplitudes from the PIV results to set the pressure boundary criteria for CFD, i.e., we chose the inlet pressure amplitude such that the axial velocity amplitude averaged over the inlet surface area was the same as in the measurements.

Simulations and meshing for the experimental models were performed using the finite element solver COMSOL Multiphysics® (COMSOL AB, Stockholm, Sweden). For the idealized models, structured meshes were generated as described in Harte et al. [47], [48]. The mesh size was chosen based on a convergence analysis, and Lagrange elements of order two and one were used for velocity and pressure (P2P1), respectively. For the time-dependent solver, the implicit backward differentiation method of variable order (between 1 and 5) was used. We chose 100 steps per oscillation period of the pressure boundary condition. To ensure that the initial transient was washed out, the inlet pressure amplitude $P_{0}$ was ramped up smoothly over the first few cycles, e.g., 16 cycles at 5Hz.

**Fig. 4:**
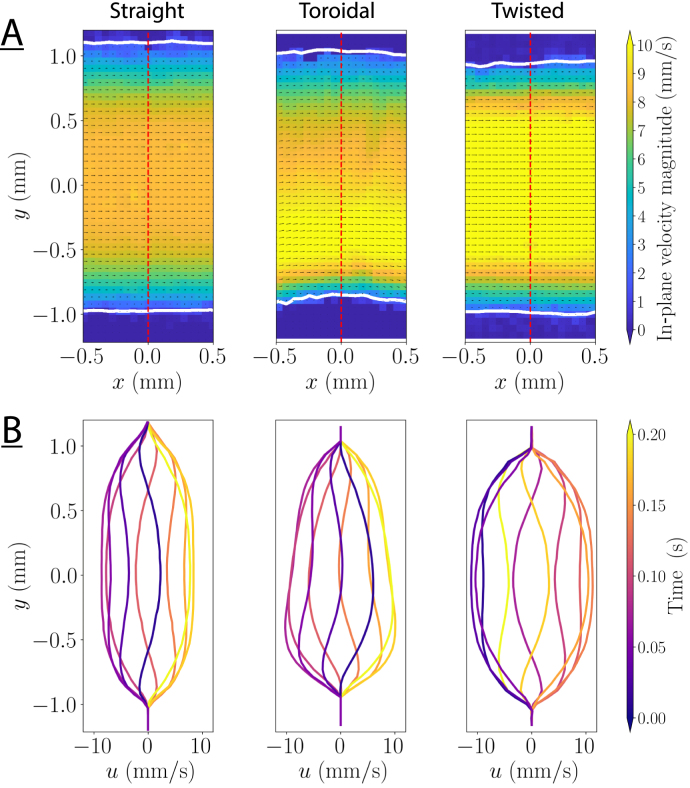
In-plane velocities in a central measurement plane for an oscillation frequency of 5Hz measured with PIV. (A) 2D flow field at peak velocity. (B) Axial velocity profile at different times over one oscillation cycle. The red line (at x=0mm in A) indicates the position where the velocity profiles were evaluated.

**Fig. 5:**
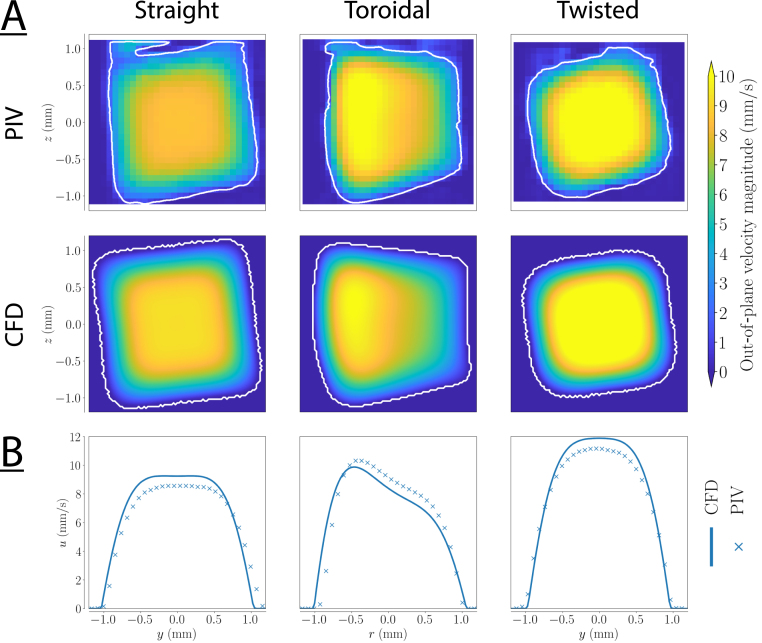
(A) Axial velocity amplitude for an oscillation frequency of 5Hz. The inner wall of the curve is to the left in the toroidal duct. (B) Cross-section of the velocity profiles at a central z-position for both the measurements and CFD simulations.

The motion of tracer particles was determined using the COMSOL Multiphysics® “Particle Tracing for Fluid Flow” interface. We used the massless formulation assuming that the tracer particles were neutrally buoyant and small enough to move passively with the local fluid velocity (Stokes number of approximately $6\cdot 10^{-5}$). To compute the Lagrangian mean velocity, the particles were released and traced for one cycle. The difference in the end positions divided by the time interval T=1/f was taken as the Lagrangian mean velocity.

#### 2.3.3 Transverse and axial flow

In the idealized models, we applied the orthonormal Frenet–Serret frame consisting of the tangent, normal, and binormal unit vectors to decompose the velocity field and defined the velocity component along the tangent as axial flow. The components in the normal and binormal directions then constitute the transverse flow [47], [48]. In the geometry-specific models, the centerlines were extracted using kinematic surface fitting [58], [59] and cross-checked with the vascular modeling toolkit [60]. As in the idealized models, the transverse plane was again defined to be orthogonal to the centerline.

## 3 Results

### 3.1 2D flow field

Fig. 4 shows an example of in-plane velocity fields measured with PIV in the middle of the geometry, which was obtained by analyzing images of the same layer from consecutive volume scans. The profiles in Fig. 4B show the axial velocity component u along the red lines at different times, and are typical Womersley profiles for low Womersley numbers (α≈4.5) in all three geometries: compared to steady Poiseuille flow, the core the profile is flatter and the velocity gradients near the walls are steeper. The axial velocity is well resolved in time and space and shows the expected behavior. In the toroidal geometry, the velocity profile is skewed towards the inner side of the curve, as will be discussed in the next section. In contrast, the profile of the twisted duct exhibits a similar velocity profile as the straight duct. This is in agreement with Kheshgi [44] and Zabielski and Mestel [61] who observed that torsion has only a small impact on axial flow. The differences in the magnitude of the axial velocity in Fig. 4 are caused by different hydrodynamic resistances and by the reconfiguration of the flow loop, as we used the same voltage amplitudes to drive the actuator pump.

**Fig. 6:**
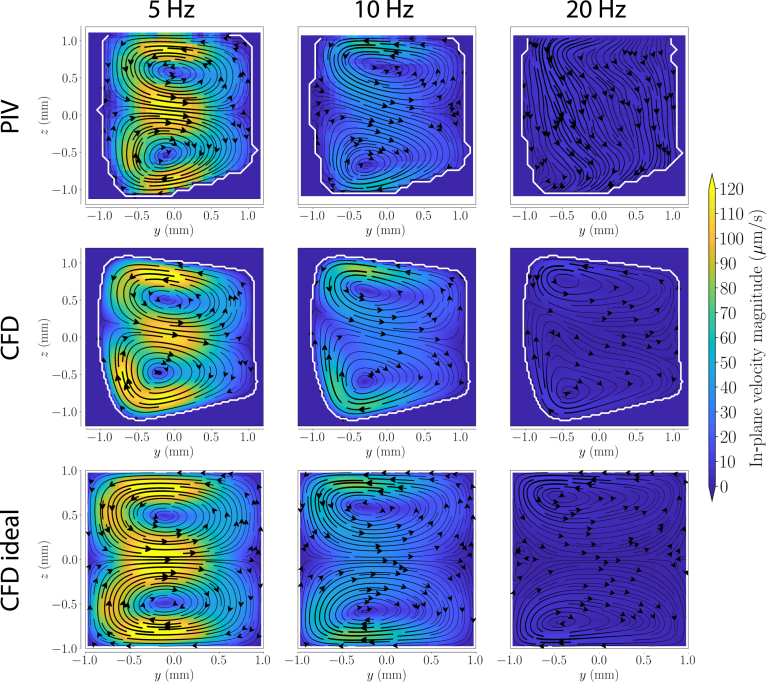
Transverse net motion (i.e., Lagrangian streaming) in the toroidal duct for oscillation frequencies of 5, 10 and 20Hz. The top row displays PIV results, while the middle and bottom rows show CFD simulations for the experimental and idealized models, respectively. The inner wall of the curve is on the left. The white lines indicate the approximate wall locations.

### 3.2 Axial flow

Extending the 2D analysis of the previous section to all the plane of the volumes provides the main axial flow in 3D. Fig. 5A shows the axial velocity amplitude in the transverse plane for an actuation frequency of 5Hz (α≈4.5) according to both the PIV measurements and geometry-specific CFD simulations. There is a small deviation in the flow amplitude between the measurements and the simulations, most likely due to either a slightly different location along the axis, or to the finite precision of the µ-CT scan. Fig. 5B shows the cross-section of the measured and simulated velocity profiles, demonstrating the quantitative agreement between CFD and PIV results. More specifically, the peak velocity is approximately 4% lower in the CFD model of the toroidal duct than in the PIV measurement for this sample, while it is 6% and 8% higher for the straight and twisted duct. Nonetheless, the axial velocity profiles obtained from the geometry-specific simulations are in good agreement with the PIV measurements. In the toroidal geometry, the maximum velocity shifts towards the inner wall of the bend, contrary to expectations from higher Reynolds numbers where the velocity profile is skewed towards the outer wall. This shift towards the inner wall is consistent with literature and was observed and predicted for low Reynolds number and high curvature [62], [63]. However, a complete explanation was missing. Our interpretation is that along the inner wall, the axial pressure gradient is steeper compared to the outer wall because the arc length is shorter than along the outer wall. Since the centrifugal forces pushing the fluid outwards are weak at low Dean numbers, the higher pressure gradient along the inner wall dominates, skewing the velocity profiles towards the inner wall [47], [64].

### 3.3 Net transverse flow

**Fig. 7:**
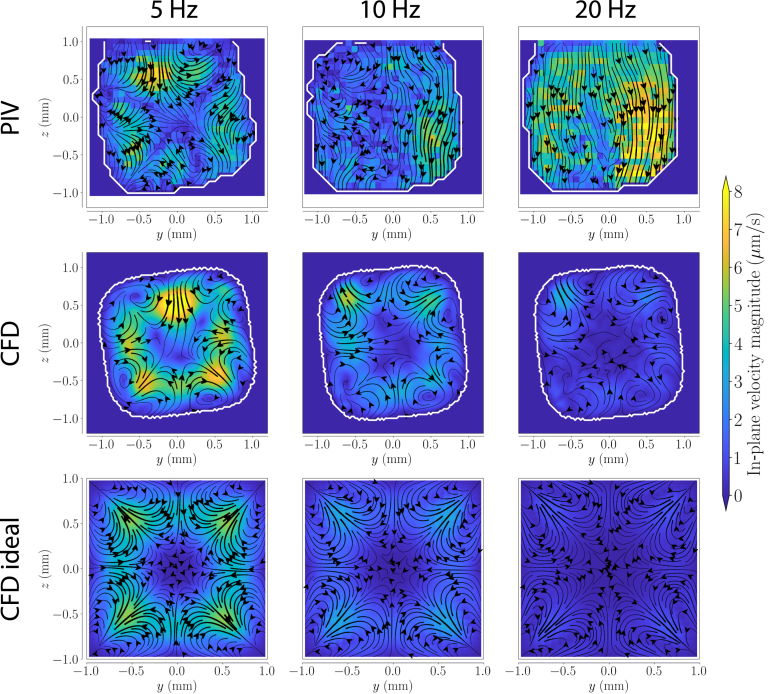
Transverse net motion (i.e., Lagrangian streaming) in the twisted duct for oscillation frequencies of 5, 10 and 20Hz. The top row displays PIV results, while the middle and bottom rows show CFD simulations for the experimental and idealized models, respectively. From the reader’s perspective, the twisted duct turns clockwise.

Now that we have confirmed that the axial flow precisely follow the theoretical expectation and the numerical predictions, we can look in to the details of the transverse flow, starting with the net motion. The net motion of the tracer particles (i.e., the Lagrangian mean velocity) in the transverse plane was quantified using the stroboscopic PIV approach (see Section 2.2.2). Fig. 6, Fig. 7 show the transverse net motion in the toroidal and twisted duct, respectively, for frequencies of 5, 10 and 20Hz. The white lines indicate the approximate wall locations. The absence of closed streamlines in the idealized twisted duct can be attributed to the absence of particle trajectories data close to its boundaries. In curved ducts, the tracer particles follow the streamlines of Dean cells (Fig. 6). The Dean flow points towards the outer wall in the ducts center and towards the center of curvature close to the top and bottom boundary of the cross-section. At higher oscillation frequencies, the vortex centers move closer to the cross-section’s top and bottom boundaries and to the inner wall. The shape of the Dean cells is consistent between the CFD and the PIV results. In particular, they both show the same asymmetry in the Dean cells caused by the specific, nearly trapezoidal, cross-section of the duct. Unlike in the idealized models, the axis between the two Dean cells is tilted in the models with the trapezoidal cross-section. In both, the tilt is more pronounced at 10Hz than at 5Hz for the measurements. Furthermore, the Dean cells are not equal in size.

At 5Hz, the transverse structures show flow magnitudes on the order of 100 µm/s, i.e., 1.6% of the axial flow. The net flow is captured accurately and quantitatively. The same holds for 10Hz, where the transverse flow amounts to 0.6% of the axial flow, i.e., approximately 40 µm/s. However, at 20Hz, the Dean cells have a velocity on the order of 0.3% of the axial flow, i.e, about 10 µm/s, based on the CFD simulation. Although the streamlines computed from the PIV results look well defined, they suggest that there is a mass source on the top of the channel, and a mass sink at the bottom, which is not physical. Therefore, we conclude that the transverse flow generated in these conditions falls below the accuracy of the proposed method. Flow ratios hereafter are given as relative to the mean axial flow amplitude.

In the twisted duct, the net motion was more than an order of magnitude smaller than in the toroidal duct (the velocity magnitude scales were reduced by a factor 15). The transverse flows caused by torsion change direction with the axial flow, and are thus largely averaged out when performing such time-averaged measurements (Fig. 7). In addition, in the twisted duct, the net transverse flow consists of complex, small-scale structures. Therefore, since the exact transverse planes visualized in the measurements is an arbitrary choice, it is difficult to perform a detailed quantitative comparison. Nonetheless, the defining structures are very similar for the measurement and for the CFD at 5Hz, with a pair of counter-rotating vortices in each corner and a net flow through the center. The magnitude of the transverse flow is also in quantitative agreement between the CFD and the measurements, and amounts to approximately 0.1% of the axial flow (6 µm/s). Because of these low net flows, the PIV analysis only reveals the strongest transverse features at 10Hz, and at 20Hz the transverse flow is lost altogether. From CFD, we expect fractions of 0.05% and 0.03% for 10Hz, and at 20Hz respectively. The net motion in the experimental model cross-section deviates strongly from that in the idealized duct. For example, at 5Hz, the strongest velocities are on the diagonal in the idealized ducts, while in the measurement they are close to the center of the side walls (Fig. 7). This highlights the need for geometry-specific strategies in complex, real-world systems.

**Fig. 8:**
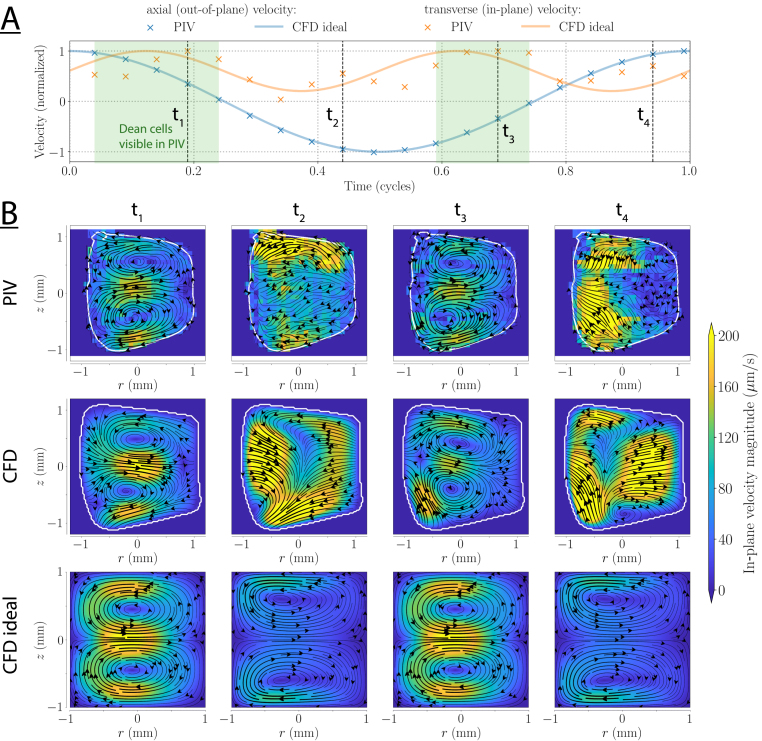
(A) Axial and transverse velocities for an oscillation frequency of 5Hz in the toroidal duct. The axial flow is shown at the center of the cross-section in relation to the transverse flow evaluated at the location of maximum transverse flow within the cross-section. To improve comparability, the location of the maximum transverse flow in the PIV was chosen at a point approximately corresponding to the simulation. Green shaded areas indicate times when Dean cells are visible in PIV. The vertical lines indicate the times at which the transverse flow patterns are shown in (B). (B) Transverse flows at different time points in one cycle. The inner wall of the curve is to the left.

### 3.4 Dynamic transverse flow

**Fig. 9:**
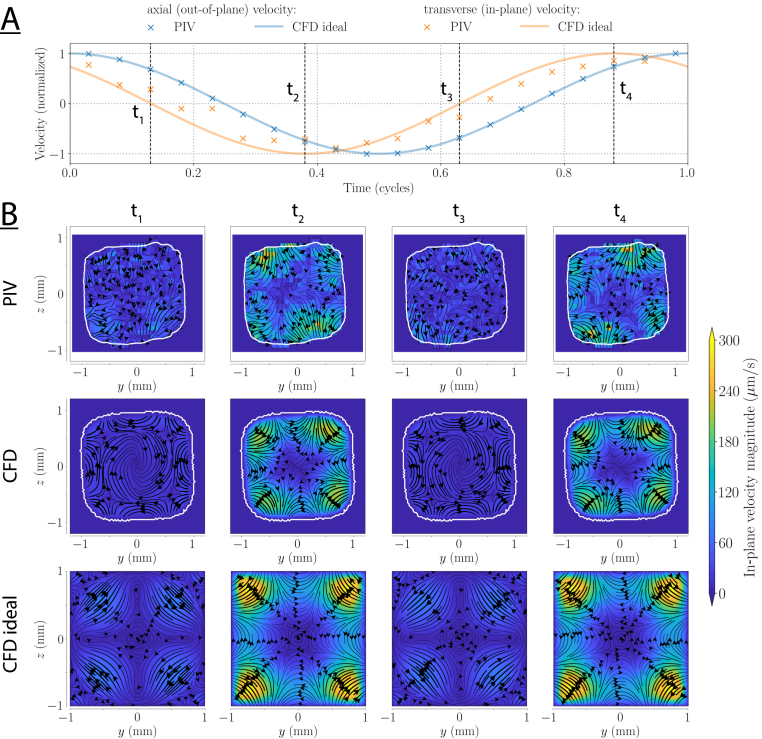
(A) Axial and transverse velocities for an oscillation frequency of 5Hz in the twisted duct. The axial flow is shown at the center of the cross-section in relation to the transverse flow evaluated at the location of maximum transverse flow within the cross-section. To improve comparability, the location of the maximum transverse flow in the PIV was chosen at a point approximately corresponding to the simulation. The sign of the transverse flow is set according to the direction of the transverse flow (i.e., clockwise/counterclockwise in the corners). The vertical lines indicate the times at which the transverse flow patterns are shown in (B). (B) Transverse flows at different time points in one cycle. From the reader’s perspective, the twisted duct turns clockwise and the axial velocity is inflowing for $t_{4}$ and outflowing for $t_{2}$.

The Lagrangian net motion, however, does not yet provide a dynamic view of the transverse particle motion within a cycle of the flow. To that end, we use the semi-Lagrangian analysis as described in Section 2.2.3. The results are displayed in Fig. 8, Fig. 9 that show the instantaneous, transverse flow pattern in the toroidal and twisted duct, respectively, at different time points within one oscillation period. The reference time for this period corresponds to the maximum axial center velocity.

For the toroidal ducts, counter-rotating Dean cells occur twice per cycle (green shaded areas) before the inflection point of the axial flow, i.e., before the point of flow reversal. The measured phase difference with the main flow is approximately 70 degrees. Note that the Dean cells rotate in the same direction for both axial flow directions ($t_{1}$ and $t_{3}$, respectively). However, irregularities in the real duct geometry produce slightly different patterns for the two axial flow directions. When the axial flow is maximal (i.e., close to $t_{2}$ and $t_{4}$), the rotating cells appear to be concealed by stronger flows. These flows increase with the axial flow and are probably geometry-induced as they appear in the CFD model as well as the measurement, but not in the idealized duct simulations. In these idealized models, the Dean cells have a phase lag of approximately 55 degrees relative to the maximum axial flow. Consequently, they reach their maximum strength before $t_{1}$ and $t_{3}$ which is a considerable difference to the measurements. We suspect that the shift in the phase lag originates from the trapezoidal cross-section of the measurement model. In contrast to the ones in the measured models, the Dean cells in the idealized model are present during the entire cycle, changing only in magnitude.

In twisted ducts, the transverse flow patterns are strongest at $t_{2}$ and $t_{4}$ (Fig. 9). The typical saddle flow structure emerges near the walls and corners at these times. Most importantly, unlike the Dean cells, the saddle flow structure changes direction for inflowing and outflowing axial velocities. The weak net flow in Fig. 7 is the residual of the near cancellation of this alternating flow. Unlike the net flow, the time-resolved transverse flow pattern is similar in the idealized and geometry-specific CFD. At 5Hz, the saddle flow structure has a phase difference of approximately 45 degrees to the axial flow in all three models. Features of the saddle flow structure are distinguishable throughout most of the cycle, even in the PIV results. Although the transverse velocities in the twisted duct are higher than the Dean flow in the toroidal duct, there is less agreement with the simulation in comparison with the other results.

### 3.5 Maximal dynamic transverse flow

**Fig. 10:**
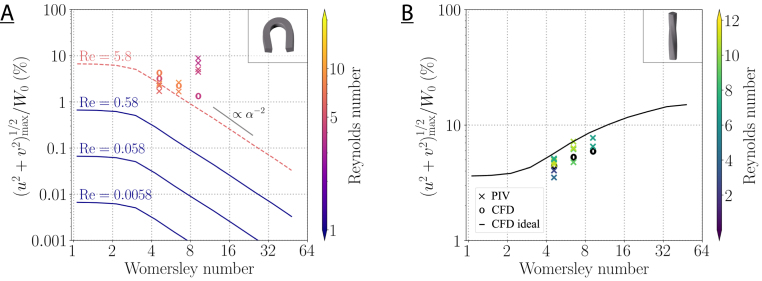
Maximal transverse flow with respect to the mean axial flow amplitude $W_{0}$ as a function of the Womersley number α for the toroidal (A) and the twisted duct (B). The crosses indicate the PIV measurements, the circles denote the simulations in the geometry-specific experimental models, and the lines show the idealized CFD results from [47]. The lines for different Reynolds numbers (0.0058–0.58) are plotted on top of each other in the right image. The dashed line for Re=5.8 is interpolated from the results at lower Reynolds numbers.

We have shown that the transverse flows can be accurately measured and match the numerical prediction made of geometry-specific mode, the net transverse flow and the dynamic transverse flow. We now assess the dependency of the transverse flow magnitude on the two relevant non-dimensional parameters of the problem: the Womersley number and the Reynolds number. Fig. 10 compares the strength of the maximal transverse flows with idealized model data [47]. We varied the flow amplitudes in the PIV measurements to test the expected transverse flow strength at different Reynolds numbers. The maximum Dean flow in the idealized toroidal duct scales with the square of the Reynolds number Re, while the saddle flow structure in the twisted duct is proportional to Re since it is a kinematic effect [46]. Thus, the curves for different Reynolds numbers collapse for the twisted duct in Fig. 10B, but not for the toroidal duct. In the PIV measurements, we successfully quantified transverse flows down to 5% of the axial flow amplitude in the twisted duct and down to 2% of the axial flow amplitude in the toroidal duct at 5Hz.

The maximum strength of the transverse flows in the twisted duct is lower than could be expected from the idealized simulation. The measured specimen has a higher torsion ($\tau =1/6\;{\text{mm}}^{-1}$) than the idealized models did ($\tau =1/8\;{\text{mm}}^{-1}$). Bolinder [46] found that the maximum strength of the transverse flows is proportional to $\tau \;d_{h}\;Re$ in twisted ducts under steady scenarios, where $d_{h}$ is the hydraulic diameter. Therefore, we would expect the measured sample to display transverse flows that are stronger by a factor of 8/6=1.333. However, the idealized square cross-section has a greater distance from the center to the corners than the real cross-section. We suspect this to counteract the change in torsion.

Within the toroidal duct, we identified the maximum transverse flows during time instances when the Dean cells were fully developed, i.e., $t_{1}$ and $t_{3}$ in Fig. 8 for the PIV measurements and the geometry-specific CFD. At 5 and 10Hz the magnitude of the Dean flows is similar to the one expected from the idealized models. Some experimental flow velocities are on the higher side of the expectations from the idealized geometries, which could be due to geometry induced flows that enhances the maximum transverse flows. At 20Hz, the PIV measurements yielded much higher maximal transverse flows than expected from the CFD results because the Dean flow could not be detected in the PIV data. In the simulations of the idealized duct, the strength of the Dean cells decreases with the frequency and with the square of the axial flow for sufficiently high Womersley numbers according to Harte et al. [47]. This is consistent with the observed features in the PIV measurements.

## 4 Discussion

### 4.1 3D-PIV system validation

Our 3D-PIV system offers several advantages. It is cost-effective, requiring only one single camera and a single optical access for the laser, in addition to one for the camera. The proposed single camera system is especially beneficial when working with small scale models, as it simplifies the setup and calibration procedure. In addition, it allows the measurement of transverse flows from a side and top view, without the need to directly capture the transverse plane, which would be challenging, especially in the case of a twisted duct. Finally, the associated optical system allows for a depth of field comparable to, or larger even, than the field of view, which allows for imaging specimens with large aspect ratios.

Nonetheless, the system also has its limitations. While the transverse flow patterns were clearly recognized in PIV analysis at low oscillation frequencies (5 and 10Hz), they were either close to or below the noise floor for 20Hz. This is attributed to the combined effect of their decreasing magnitude and the increasing experimental noise at higher frequencies. At higher frequencies, the transducer produced a lower flow velocity amplitude and smaller particle displacement, which is suboptimal for PIV analysis. At the same time, higher frame and scan rates are required to obtain a sufficient number of frames per period. To compensate for the shorter exposure time, we opened the aperture more, resulting in less depth of field in the scan direction. This is the main technical limitation of the study.

The stroboscopic PIV analysis was implemented for transverse net motions, therefore no axial net motion was evaluated since it was outside the scope of this study. While this axial net flow is probably not substantial in the toroidal duct, see e.g., Zabielski and Mestel [61], the twisted duct may have an axial net flow component. The method, however, can be readily extended by performing the stroboscopic PIV analysis in the orthogonal planes aligned with the channel axis. Furthermore, the SNR of the method can be further increased by performing the analysis at each recorded phase in the driving cycle and averaging the result.

We tried two other approaches to obtain the instantaneous time-resolved transverse velocities, which, in our case, gave a lower SNR than the Semi-Lagrangian PIV: In the first approach, we analyzed the xy- and xz-planes separately and then combined the v and w velocities obtained from the respective planes. The second approach was to run a PIV analysis on the measurement plane and then obtain the out-of-plane velocity w from the divergence of the velocity field ∇u=0 by integration. As the divergence has more noise than the velocity components, the SNR was found to be unsatisfactory.

### 4.2 Sample imperfections and net transverse flow

We have observed large differences between the transverse flow patterns in idealized geometries and in the measured samples. We attribute these differences to uneven sample walls and, more importantly, to rounded corners. However, these differences are not artifacts since they are recovered by the geometry-specific CFD simulations. For example, in the twisted duct, the observed flow through the center probably results from variations in the twist angle and the curvature of the centerline itself. In addition, the tilt of the axis between the Dean cells in the toroidal duct is not present in the idealized model. Such effects are particularly relevant for physiological structures such as the cochlea, which are intricate geometries with torsion, curvature, and varying cross-sections.

We suspect that the transverse net motion in the twisted duct originates from the Stokes drift, as the Lagrangian mean velocity significantly exceed the corresponding Eulerian mean velocities (in the simulations). The Stokes drift arises from the oscillatory characteristics of the saddle flow structure and local gradients in the axial velocity. During the first half of the oscillation cycle, the particles move in one direction, only to change direction during the second half. Since the initial position of the particle is not exactly the same during these two halves, the particle experiences different forces from the local flow field. This mismatch causes a net drift also known as Stokes drift [65] which is inversely proportional to the oscillation frequency in cochlear ducts [29]. Accordingly, we also observed that these net motions decreased with the oscillation frequency in the idealized geometries.

### 4.3 Alignment and dynamic transverse flow

Notably, the Dean cells in the measured models were only observed in two short time intervals during each cycle, and they were even less pronounced at higher frequencies in CFD. This may be due to three reasons. First, the geometric differences between the idealized models and the experimental models can lead to additional flows, that are overlaid with the transverse flows. Second, it could be due to their sensitivity to the orientation of the transverse plane; even a small deviation in the plane orientation during post-processing causes a part of the axial flow to be inaccurately defined as transverse, resulting in a bias of the actual transverse flow. Third, the Dean cells may still be developing in the experimental models and therefore may still be subjected to entry length effects.

The transverse flow presented for the twisted duct are averaged over a short axial distance. However, the twisted duct displays a continuous twist, and this average may thus impact the reconstructed flow field. The quantitative comparison with the geometry-specific CFD model suggests that the influence is limited. Nonetheless, implementing corrections such as a rotating reference frame to account for the rotating cross-section could improve the results for the twisted duct, potentially down to the order of 0.1% with respect to the axial flow.

### 4.4 Potential applications and implications

The proposed measurement technique allows for investigating periodic flows in models of micrometric to millimetric systems that cannot be scaled up because of their multi-physics properties. This includes flows induced by membrane motion as, e.g., in the inner ear [66], [67], [68], flows with solid particles or cells, e.g., in organs-on-chips [12], [69], [70], and cilia-driven flows, e.g., in the models of trachea [71], [72], or amphibian skin [73], [74]. Other possible applications include microfluidics [75], [76] and in vitro models of millimeter-sized blood vessels, which are often curved and twisted, e.g., umbilical veins and helical umbilical arteries [77], [78], superficial femoral arteries [31], and the circle of Willis [79].

The observed cross-sectional Lagrangian streaming may be of interest for mixing and mass transport in small enclosed systems such as the cochlea. Streaming velocities were found to decrease with oscillation frequency in both curved and twisted channels. This suggests that mass transport induced by the geometry may be more prominent at low stimulation frequencies. This is in line with Edom et al. [29], who characterized steady streaming in the cochlea which, among others, originated from fluid structure interaction with the vibrating basilar membrane. They also indicated that steady streaming is mainly relevant to the low-frequency hearing process. Frequencies in the infrasonic regime (i.e., below 16Hz) are not considered audible, they can, however, still lead to fluid motion in the cochlea. In addition to passive diffusion, steady streaming could help transport ions and metabolites throughout the cross-section. This is important as the structure that secretes ions into the endolymph is located at the outer wall of the spiral duct, from where they need to be transported to the surrounding tissues and sensory hair cells.

## 5 Conclusion

We have developed a single-camera, scanning 3D-PIV system for small-scale oscillatory flows, resolving subtle transverse flows at a few percent of the main axial flow amplitude. The oscillatory axial flow amplitudes were varied between 3 and 8 mm/s. We applied the system to three millimetric geometries: a straight square duct, a twisted square duct, and a toroidal duct with a trapezoidal cross-section. Curvature and torsion lead to time-resolved transverse flows (Dean cells, saddle flow structure) and time-averaged net motion. The net motion in the toroidal duct, i.e., Dean flow, was stronger and thus easier to visualize than the one in the twisted duct. The detected net motion was approximately 1% of the axial flow in the toroidal duct and about an order of magnitude lower in the twisted duct. We measured time-resolved transverse flows down to 5% of the axial flow amplitude in the twisted duct and down to 2% of the axial flow amplitude in the toroidal duct at 5Hz. The proposed 3D-PIV system will be beneficial for true-scale measurement of transverse flows in minichannels to microchannels in industry and in-vitro models of complex biological systems, e.g., involving solid particles, cells, or cilia.

## CRediT authorship contribution statement

**N.C. Harte:** Writing – review & editing, Writing – original draft, Visualization, Validation, Software, Methodology, Investigation, Formal analysis, Data curation, Conceptualization. **D. Obrist:** Writing – review & editing, Validation, Supervision, Methodology, Conceptualization. **M. Versluis:** Writing – review & editing, Validation, Resources, Project administration. **E. Groot Jebbink:** Writing – review & editing, Validation, Methodology. **M. Caversaccio:** Writing – review & editing, Resources. **W. Wimmer:** Writing – review & editing, Visualization, Validation, Supervision, Resources, Project administration, Methodology, Funding acquisition, Conceptualization. **G. Lajoinie:** Writing – review & editing, Writing – original draft, Visualization, Validation, Supervision, Software, Resources, Methodology, Funding acquisition, Formal analysis, Conceptualization.

## Declaration of competing interest

The authors declare that they have no known competing financial interests or personal relationships that could have appeared to influence the work reported in this paper.

## Figures and Tables

**Fig. A.11:**
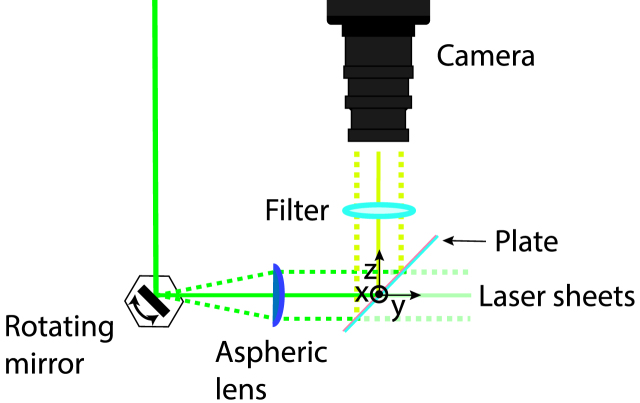
Setup for calibration.

**Fig. A.12:**
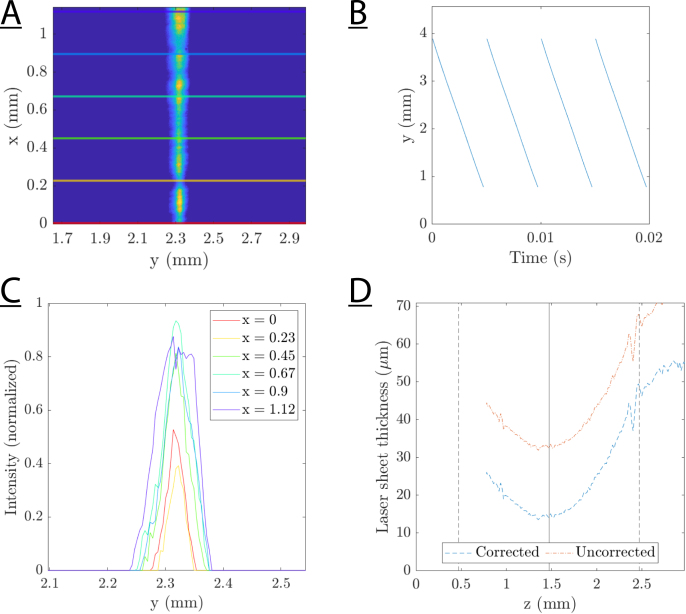
Laser sheet characteristics. (A) Image of the laser sheet as captured by the high-speed camera. (B) Position of the laser sheet as a function of time. (C) Intensity over the image of the laser sheet at different widths x (in mm) across the measured plate. (D) Laser sheet thickness before (red) and after (blue) correction with the PDMS layer thickness. The solid vertical line indicates the z-position where focus is best, while the dashed lines indicate the borders of the test section.

**Fig. A.13:**
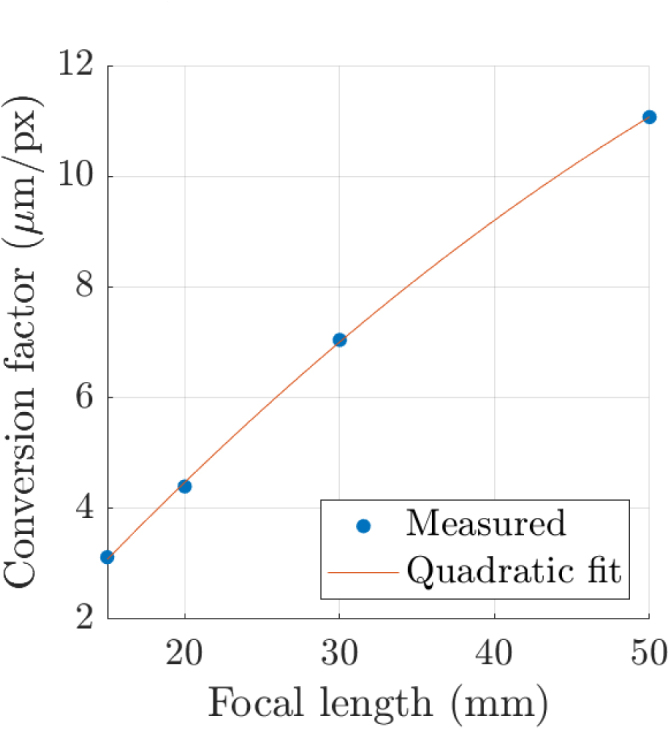
Conversion factor from pixels to micrometers for different focal lengths of the frontal lens.

**Fig. B.14:**
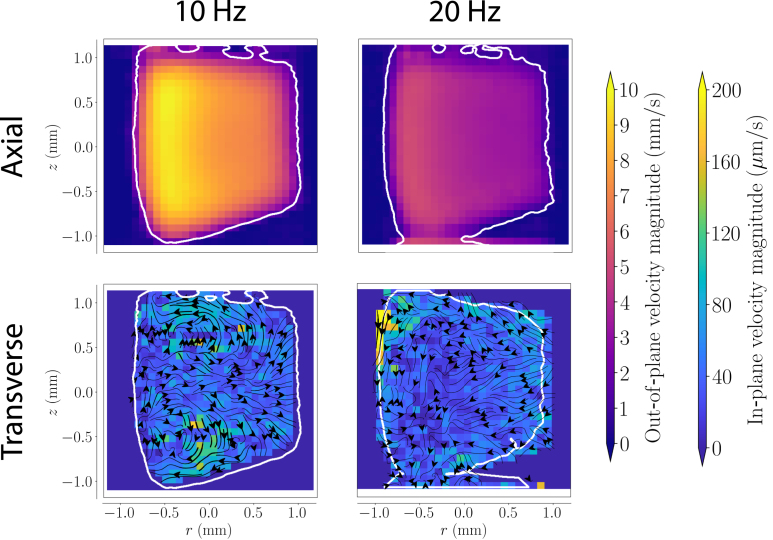
Dynamic axial (first row) and transverse flow (second row) in the toroidal duct.

**Fig. B.15:**
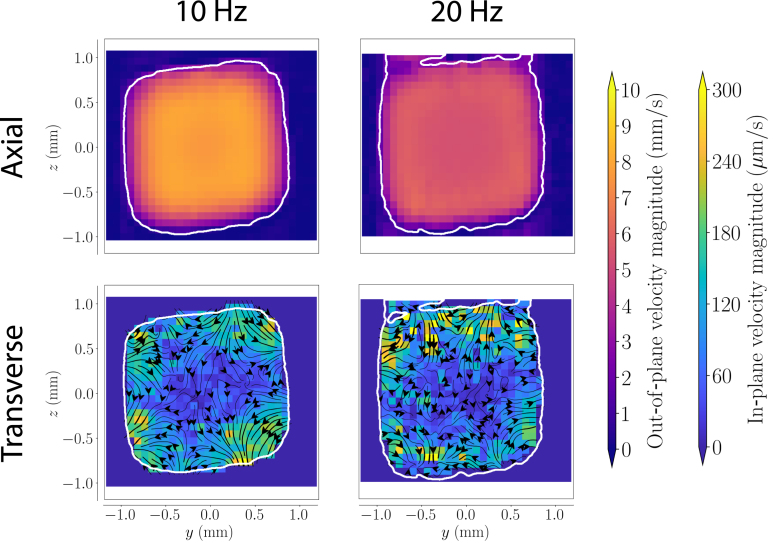
Dynamic axial (first row) and transverse flow (second row) in the twisted duct.

## Data Availability

Data will be made available on request.

## Appendix A System calibration

### A.1 Laser sheet thickness

To characterize the laser sheet, we used a glass plate with a thin layer of PDMS mixed with fluorescent Nile Red. The plate was fixed at a 45° angle to the laser and camera (Fig. A.11). The plate was scanned with the laser sheet, as in the measurements, and the camera consequently captured images at different heights z and positions y along the plate. We used a laser scan rate of 200Hz and a camera frame rate of 40  kfps. Fig. A.12B shows the height of the laser sheet as a function of time, which reveals the sawtooth function used to control the laser height. Note that the height corresponds to the y-coordinate as the plate is at a 45° angle to the laser and camera. Fig. A.12A shows the line of the laser sheet at one height, and Fig. A.12C shows the intensity profile over it at different x-position. The beam is Gaussian and its width was defined as the 1/e width:

(A.1) $$\delta_{1/e}=2\sqrt{2}\sigma ,$$

where σ is the standard deviation from a Gaussian fit of the intensity profile in Fig. A.12C.

The beam width is larger than the laser sheet thickness, because of the non-zero layer thickness of the PDMS. We measured the PDMS layer thickness $\delta_{PDMS}$ with confocal laser scanning microscopy to be 13±0.7  µm. To correct for this, the laser sheet thickness $\delta_{l}$ was calculated as

(A.2) $$\delta_{l}=\delta_{1/e}-\sqrt{2}\delta_{PDMS}.$$

We used the median beam width over the x-direction. Fig. A.12D shows the resulting laser sheet thickness and the original beam width as a function of the height. The minima correspond to the layer where the focus of the camera system is best and the measured thickness increases below and above due to focusing. The laser sheet thickness was thus approximately $\delta_{1/e}=31.9$  µm before correction and $\delta_{l}=13.5\pm 0.9$  µm after correcting for the thickness of the PDMS layer.

### A.2 Scanning rate and inter-slice distance

We used a 1951 USAF resolution target, which contains line pairs of different sizes, to calibrate the PIV system. We recorded the target for different focal lengths of the reversed macro lens. The resolution was interpolated quadratically between the measured focal lengths to determine their dependence (Fig. A.13). In the PIV analysis, we then used the interpolated function to related the focal lengths set in the measurements to the corresponding pixel resolution.

The different sawtooth function amplitudes, i.e., laser amplitudes, scale linearly with the distances between the captured layers in the z-direction. The total scan height per amplitude was approximately 3.75  µm/mV. For example, for 200 layers and 800  mV, the median slice thickness was approximately 15.09±1.66  µm. This leads to a spatial imaging resolution of approximately 5×5×15  µm for a typical focal length of 22mm.

## Appendix B Dynamic axial and transverse flows for higher frequencies

Fig. B.15, Fig. B.14 show the time-resolved axial and transverse velocities in the twisted and toroidal duct, respectively, at one time point. The axial velocity is shown at its maximum. The transverse velocities are shown at the time when they are most developed.
